# Ten simple rules for early-career researchers supervising short-term student projects

**DOI:** 10.1371/journal.pcbi.1013690

**Published:** 2025-11-18

**Authors:** Rebecca M. Crossley, Philip K. Maini

**Affiliations:** University of Oxford, Oxford, United Kingdom; University of Virginia, UNITED STATES OF AMERICA

## Abstract

Supervising a short-term research project at undergraduate or master’s level is a rewarding yet complex responsibility that extends far beyond subject expertise. It offers supervisors the opportunity to inspire the next generation of scientists, while providing students with a crucial platform to develop research skills, academic identity, and resilience. However, short-term research projects often come with challenges, including varying levels of student motivation, limited time frames, and the need for intensive skill development. Effective supervision can enhance student learning, foster independent thinking, and improve both the quality and impact of student work, while also contributing to a positive and inclusive research culture. In this article, we present ten simple rules to guide supervisors, particularly those with limited supervision experience, such as early-career researchers, in supporting undergraduate and master’s students through their research journeys. These rules emphasise balancing project requirements with student interests, managing scope, fostering community integration, promoting open science practices, and providing structured yet flexible guidance. By adopting these practical strategies, supervisors can create a more productive, supportive, and enriching research experience for both students and themselves.

## Author summary

Supervising undergraduate or master’s research projects is a vital aspect of academic life, offering opportunities for both student development and supervisor growth. For many early-career researchers, it marks the first chance to mentor a junior colleague. However, short-term student projects often present unique challenges: students may be balancing coursework, lack prior research experience, or feel uncertain about independent work. Effective supervision requires more than guiding the technical aspects of a project; it involves fostering critical thinking, resilience, and professional skills that students will carry forward into their careers. This article presents ten simple rules designed to help new supervisors navigate these challenges. The rules emphasise practical strategies such as aligning project goals with student interests, managing project scope, integrating students into research communities, introducing open science practices early, and supporting both the technical and emotional aspects of research. Drawing on educational research and mentoring experience, these guidelines aim to make supervision more effective, inclusive, and rewarding. By adopting these approaches, supervisors can learn skills to help guide students toward achieving their research goals, minimise common frustrations, and nurture the next generation of researchers.

## Introduction

Supervising an undergraduate or master’s research project can be one of the most fulfilling steps in an academic’s career and is an important part in building supervision experience for young researchers. It might be the first opportunity an early-career researcher is given to supervise someone more junior than themselves, and it provides an opportunity to inspire others to pursue a career in academia. For the student, research provides an opportunity to build their identity in academic science, trial the working environment and develop their resilience when problem solving, as well as improve a variety of soft skills including communication, presentation and more [1,2].

Working with undergraduate and master’s project students involves a dynamic and often complex relationship between the supervisor and student, which can be highly beneficial but also challenging [1,3–7]. While some projects are driven by student interest, others are mandated by taught courses, leaving some students unmotivated or reluctant to begin. Even enthusiastic students often come unprepared or ill-equipped with the necessary time management skills to handle a short-term project alongside lectures, coursework or other end-of-year distractions. As such, a wealth of investment by the supervisor into the student’s training is often required before they are comfortable researching independently.

Hence, supervising a short-term research project, although rewarding, is a complex task that requires more than just subject expertise. Effective supervision enhances student learning, fosters independent thinking, and can significantly improve the quality and impact of student work [1,8]. Adopting thoughtful strategies can reduce time pressures and prevent common frustrations for both the student and the supervisor, leading to more productive, collaborative relationships. In turn, this supports a more inclusive and positive research environment, which helps sustain the future pipeline of researchers and professionals in the field [9–12].

While supervision refers to guiding a student through the successful completion of a specific academic project, a good supervisor will also act as a mentor–focusing beyond the immediate research task and onto the students’ broader personal and professional development. Although this article follows the ‘ten simple rules’ format and is not intended as an exhaustive or evidence-based review, it draws from both experience and a wealth of educational literature [3,8,13,14]. In particular, these upcoming rules align with several of the ‘salient practices’ of undergraduate research mentors [8,13]. For example, we share an emphasis on celebrating student achievement, scaffolding writing, maintaining clear expectations, and creating a sense of research community. However, our rules differ slightly in emphasis, focusing on short-term undergraduate or master’s research project or dissertation specific supervision (such as scoping and tracking) and less on creating additional, long-term mentoring networks. This is complementary to the existing literature on mentorship–offering actionable advice for early-career researchers and first-time supervisors who may not yet have the capacity to provide broader mentoring structures but still play a vital role in fostering student growth.

As such, this article is aimed at early-career researchers with limited supervision experience supervising first-time researchers at undergraduate or master’s level, where understanding how to support students effectively can benefit both the student’s development and the supervisor’s own professional growth. As such, the ten simple rules that follow aim to support new supervisors with practical, research-informed strategies for effective and inclusive supervision, and to provide suggestions for ideas that supervisors may not have previously considered. Where not otherwise stated, the advice in this article does not therefore pertain specifically to students or supervisors at a particular career or level of experience–instead, the rules presented here may be beneficial more widely to any potential supervisor, irrelevant of their career stage or mentoring context.

## Rule 1: Consider the project requirements as well as the student’s strengths and passions

A good research project is not just interesting–it is achievable within the allotted time frame. As the supervisor, it is simple to start by thinking about what excites you, is within your knowledge, expertise and remit, and what work you would ideally like to see done. However ultimately the chosen project needs to be tailored to the student’s background, goals, and capacity. For example, it is good to know what the student hopes to gain from the project: would they like to learn new skills (like coding in a different language) or build on those they have already started to develop in lectures? Aligning the project with the student’s strengths can boost confidence, performance and productivity, but offering space to tackle weaknesses can be equally valuable.

For short-term projects it is especially important that supervisors carefully balance coherence with guiding students towards tasks that employ their strengths and address weaknesses, in order to see both progress and growth during the allotted time. One way to do this might be to focus the main topic of research comfortably within the student’s skill set, but pose extension questions requiring the use of unseen material or new theory. A well-matched project can motivate the student, support their development, and form meaningful research outcomes [13,15–17].

Particularly in interdisciplinary fields like computational biology, it is important to clarify the focus early: is it theoretical, computational, or biological? The choice should align with the student’s interests and the project’s assessment process–something that is often challenging since relevant expertise within one department is limited. As such, new supervisors must take responsibility for gathering and understanding the necessary information regarding project assessment by contacting the department themselves and ensuring that the project is sufficiently foundational for assessment by non-specialists, which may mean prioritising clarity and solid methodology over groundbreaking results. The goal is to design a project that is robust, assessable, and rewarding for the student.

## Rule 2: Define the scope – then halve it!

Students are often ambitious, and early-career researchers are no different. However, short-term research projects (especially undergraduate ones) have real time constraints. If the initial scope feels “about right”, it is probably too big. It can be inspiring to discuss the wider scope of the work, but it is imperative that a supervisor tries to maintain the student’s focus throughout the project. To do this, it is best to keep the core goal clear and simple. If the student gets further–great! But avoid designing a project that needs everything to go right at every step. Instead, aim for a project with modest scope that allows for extension or simplification as needed, starts at a level the student understands, draws on skills they know or can quickly pick up, and has the possibility of producing valuable results within the allotted time frame [15].

At undergraduate level, this means focusing on developing foundational research skills, reproducing results in the literature and demonstrating communication skills–jobs which are hopefully reminiscent of your own first research tasks, and everyday checks you still carry out today. At master’s level, the project should develop more depth, critically engage with the literature and there is a greater expectation of novelty and justification in the work. Having a focused and achievable project in either case allows students to explore deeply and finish strong.

Effective mentoring therefore hinges on establishing and maintaining mutual expectations between the mentor and mentee–especially with students new to research [18,19]. From the outset, supervisors should openly discuss the project goals, time commitment, and potential challenges. Try to work with the student and support them to learn their own best ways of working, for example researching during the hours of the day they are most focused and effective. To prevent student disappointment and supervisor frustration when progress does not align with initial ambitions, it helps to set clear expectations (for example in a project plan) which can be revisited during regular check-ins [17]. This not only helps students stay on track, but provides clear guidelines for what success looks like.

## Rule 3: Schedule regular meetings – and stick to them!

Throughout the literature, the benefits of regular meetings have been repeatedly highlighted [20,21]. So, even if your student seems independent, do not skip regular meetings. For full-time projects (such as master’s by research projects) weekly or fortnightly check-ins provide structure, help catch problems early, and signal that the work the student is doing matters to you. For part-time research (common during term time), meetings can occur less frequently, but early-stage meetings should be more regular to support onboarding and build confidence. For undergraduate students, or taught master’s students continuing research into the holiday, more regular meetings should be scheduled in the periods when conflicting interests (such as lectures) calm down. As an early-career supervisor, try to remain focused but friendly–ask how their week is going, review their progress, troubleshoot any challenges, and clarify the next steps before they leave. Turning up week in, week out helps the student realise that you are a reliable role model that cares, as recent work showed that much of a supervisor’s role actually involves providing emotional support to students during their project.

Regular meetings are also a good way to introduce students to the wider research landscape. Whilst it has been shown that one-to-one meetings have the highest impact on the students [2] and should certainly be prioritised, if you are supervising multiple students working on the same/similar topics in other projects, or there is another researcher in your group with more applicable skills or experience in part of the project, consider group meetings to bring people and expertise together and encourage collaboration [16]. During these meetings, some supervisors find it helpful to use shared documents to track goals and share material, which in turn helps students develop accountability for their work. These regular check-ins are where much of the actual supervision happens, and consistency builds trust.

## Rule 4: Integrate the student into the wider research community

Even short-term projects are more rewarding when the student feels part of something bigger. Throughout their project, it is important to encourage the student to attend seminars, join in with lab meetings, or share a quick summary of their work at a student conference or department poster session. These moments allow students to see their project in context and gain confidence speaking about it–if you can attend too, then even better! Where appropriate to their skill level and possible with respect to time, it may also be beneficial to introduce students to key research tasks such as peer review, funding application processes and learning to connect with other academics to build their network [8,14,16,22,23]. For example, introducing a new researcher to peer review requires careful scaffolding and provision of exemplar materials to ensure that they understand both the expectations from the journal itself and of peer review in general. Do you both have the capacity for this? It requires teaching the student to first check if the work is correct, before looking for sufficient advancements in the methodology or findings. After this, they need to ensure the work fits the journal remit and also that they understand the purpose of the work–this is a lot of responsibility for just one review, for example!

Furthermore, engaging the students in informal activities outside work time can help develop the student’s interest in the research itself [11,24]. Some supervisors arrange a day out, plan sporting activities or even just go for lunch together. A sense of community can transform the research environment from an isolated place into a collaborative experience.

## Rule 5: Develop their skills, not just their output

Arguably, developing the skills of the student is the primary responsibility of a supervisor [6,20,25]. It can be easy to focus on the deliverables–final reports, code outputs, or figures–but the real success of a research project lies in skill development. Undergraduates, and even some master’s students, often have varying levels of expertise with software, coding languages, or analysis tools required for their project [6,7,22]. Whether it’s learning to code, write scientifically, or read papers critically, making sure students leave with a personalised toolkit they can carry forward is essential.

Rather than expecting students to learn on their own, assess their skill level early on and offer targeted support, such as access to lecture notes in courses they have not taken or a new textbook recommendation. Another way to assess skills might be getting a student to review and present a relevant paper. Asking the student questions about the method and results interpretation, as well as possible next steps, will help gauge the student’s level of understanding. A supervisor could point their student to online tutorials, write short introductory guides or show them how to complete simple warm-up tasks to get started. Technical training does not have to be exhaustive, but it should be purposeful. Talk to them about the transferable skills they are developing, and remind them that even if the project does not yield “publishable” results, a student who develops and grows their research skills will certainly be a successful result itself.

## Rule 6: Introduce good practice early

One key example of the skills students often need to develop are good research practices. Namely, there is a reproducibility crisis in research, so teaching open science practices, introducing version control (*e.g.*, Git), code sharing, and data management at the very start of a project helps students build good habits [26–29]. Providing course recommendations for this can be one easy way to help. Learning good organisational skills for a research project [30,31] (such as examples of how to use lab books, keeping well-documented Jupyter notebooks with the associated equations, headings and background information, as well as recording the parameters used to create a figure or sharing annotated datasets) can reinforce the importance of transparency and will make writing up the final report a lot easier, too. These practices not only build student confidence in research integrity but prepare them for collaborative science.

## Rule 7: Balance guidance and independence

It is tempting to over-direct student research, especially when under time pressure. However, one of the most valuable things that students learn during a research project is how to think independently. Often, this means taking a more hands-on role in supporting the student at the start when they are new to the methods, the context, and the research environment in general, by providing scaffolded targets and assignments between meetings to start with, but allowing more independence towards the end of the project [25]. Instead of solving problems for them, ask open-ended questions, such as: “What do you think might be going wrong?” or “What would you try next?” Try to make yourself approachable and available for help as it is needed (even outside scheduled meetings where possible) [17,22,25], but resist the urge to micromanage their every step. A good supervisor recognises a student who needs more guidance, whilst providing others with the space to make decisions (and mistakes) within a supportive framework [8].

## Rule 8: Normalise uncertainty

Many students feel like they are the only ones struggling–especially when things do not work. It is important to normalise setbacks, confusion, and even feeling like an imposter, making it clear that these are common experiences [32–34]. Share stories (yours or others’) that demystify research as a messy, human process, and emphasise that not knowing something is not a weakness, but a starting point for learning. Encourage students to ask questions, and suggest they read articles highlighting the importance of being unsure [34–36]. Supervisors should be honest that they do not have all the answers, they are learning together, and sometimes a suggestion might not work. This transparency helps normalise uncertainty and fosters a culture of collaborative problem-solving. Students should understand that the supervisor’s expertise lies in recognising what is likely to work, distinguishing between a mistake versus a flawed method, and guiding the project to stay on track.

As such, the supervisory process is most effective when feedback flows in both directions, so it is important to create a space where students feel comfortable asking for help, expressing confusion, or suggesting alternative directions. One way to do this can be by regularly asking questions like, “What is something you are stuck on?” or “Is there anything you would like me to explain differently?” Creating a culture where students feel safe asking questions and admitting when they are stuck will not only help their project, but can help reshape their entire relationship with science.

## Rule 9: Provide structured support for writing

Writing up results is often the most daunting part of the project for students, especially if it is their first experience with academic authorship. To help students succeed, supervisors should introduce and reinforce the basics of academic writing early, including clear naming of figures, proper equation labelling, consistent citation styles, and maintaining a logical flow. One practical way to do this might be tasking the student to identify structural themes from similar papers, or practice introducing known concepts or definitions in the style of their thesis. These conventions are not always intuitive, and failing to follow them may result in lost marks during assessment. Supervisors should encourage and guide students to use appropriate writing tools, such as Microsoft Word or 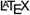. As such, templates or examples of your own work or anonymised previous student work can serve as useful references, providing a clear standard for formatting and style, particularly if they reflect the expectations of the course or institution. Discussions should be had regarding the transparent and appropriate use of generative artificial intelligence and large language models during the writing process [37,38], and examples of software to be used for figure editing should be suggested, such as BioRender, Keynote or Inkscape.

The writing process can be scaffolded with intermediate goals–such as a project outline by week three, a draft of the methods section by week six, and so on–allowing students to develop their writing skills gradually, reducing anxiety and avoiding the last-minute rush [8]. Just make sure to prepare your own calendar in advance to ensure there is time to provide relevant and timely feedback for the student, too. Throughout this process, supervisors should emphasise that good scientific writing is a skill that takes time to develop, involving many drafts and revisions. Examples of this can be reassuring, too. Creating a supportive environment where students can ask questions, receive feedback, and improve their work helps them build confidence and ultimately produce a polished, coherent final report.

## Rule 10: Celebrate little wins

Research can be a slow and uncertain process–especially for students encountering it for the first time. Days (or weeks) may pass without tangible results, which may undermine motivation or confidence. Therefore it is essential to acknowledge and celebrate small wins along the way [16]. Providing positive reinforcement, a “well done”, some feedback or support can all ease anxiety, lack of self-esteem and make the research process less stressful and nerve-wracking for the student [17]. Small wins can take many forms: finalising a figure, understanding a challenging concept, or successfully debugging a piece of code. These may not be major milestones for the supervisor, but for a first time researcher, these can be pivotal moments that build momentum and self-belief.

Celebrating does not have to be elaborate. A few words of genuine praise, a quick message of encouragement, or even noting progress during a meeting can go a long way, and have been shown to be integral to achieving high student satisfaction and success [17,23,39]. You might even consider setting up a shared document or board where you and the student can track and reflect on these accomplishments. Some supervisors do a “weekly win” or send Slack or email reactions to acknowledge progress. Taking time to recognise these small achievements will reinforce a growth mindset in a student, build a positive research culture, and help students stay engaged through the inevitable challenges of research. After all, confidence is built one small win at a time.

## Conclusion

In this article, we have presented ten simple rules for an early-career researcher supervising a short-term master’s or undergraduate research project for the first time–recognising, of course, that no project is straightforward and that every student has their own strengths, challenges and individuality [40]. These rules aim to provide the student with a strong foundation in research skills and practices, whilst offering a flexible framework to support them. Many of these principles extend beyond the scope of short-term projects and are applicable to mentoring more broadly, where early-career researchers and faculty play a crucial role in shaping the scientific experiences of students–often influencing whether they pursue further education, research, or science in general [12]. By demonstrating good practices, fostering collaboration, embodying patience and compassion, supervisors can do more than just guide a project: they can cultivate the next generation of thoughtful, capable and successful researchers, building relationships based on trust and respect [19].
